# Rare presentation of intractable tuberculous panophthalmitis with intraocular and intraorbital abscesses: a case report

**DOI:** 10.1186/s13256-017-1353-9

**Published:** 2017-07-04

**Authors:** Sutasinee Boonsopon, Nattaporn Tesavibul, Mongkol Uiprasertkul, Supinda Leeamornsiri, Pitipol Choopong

**Affiliations:** 1 Department of Ophthalmology, Faculty of Medicine, Siriraj Hospital, Mahidol University, 2 Wanglang Road, Bangkoknoi, Bangkok 10700 Thailand; 2 Department of Pathology, Faculty of Medicine, Siriraj Hospital, Mahidol University, Bangkok, Thailand; 3 Department of Ophthalmology, Faculty of Medicine, Thammasat Hospital, Thammasat University, Bangkok, Thailand

**Keywords:** Tuberculous panophthalmitis, Tuberculous orbital abscess, Extrapulmonary tuberculosis

## Abstract

**Background:**

We report a rare presentation of extrapulmonary tuberculosis.

**Case presentation:**

A 29-year-old Burmese woman with human immunodeficiency virus infection and known pulmonary tuberculosis who had been treated for 5 months presented to our hospital with unilateral progressive painful visual loss of 1 month’s duration. She was diagnosed with tuberculous panophthalmitis with subretinal and intraorbital abscesses, conjunctival abscess, and extraocular muscle tuberculoma. The diagnosis was confirmed by a conjunctival pus swab with a positive result for acid-fast bacilli and a positive result for a mycobacterial culture. There was high suspicion of multidrug-resistant tuberculosis. Despite receiving ongoing aggressive treatment with conventional antituberculous medications, this patient required subtotal orbital exenteration to control her infection and prevent further progression. Second-line antituberculous medications were added to the first-line therapy, with satisfactory results achieved.

**Conclusions:**

Tuberculous panophthalmitis with intraocular and intraorbital abscesses is a rare presentation of extrapulmonary tuberculosis. Patients who do not respond to first-line antituberculous therapy might be infected with either single-drug or multidrug-resistant *Mycobacterium tuberculosis*. Patient compliance is one of the key factors that can alter the course of treatment. Careful patient monitoring can improve disease progression, outcome, and prognosis.

## Background

*Mycobacterium tuberculosis* (Mtb) is an obligate pathogenic bacterial species in the family Mycobacteriaceae. Inhalation of this organism causes pulmonary infection. Hematogenous spread as a result of a ruptured pulmonary granuloma leads to multiple organ involvement, including the eye [1]. Failure to identify tuberculous mycobacterial ocular infection can lead to permanent visual damage, with uncontrolled infection potentially resulting in death [2]. We report a rare and serious case of a patient with extrapulmonary tuberculosis (EPTB) who presented with tuberculous panophthalmitis with intraocular and intraorbital abscesses, despite ongoing aggressive treatment with conventional antituberculous therapy (ATT).

## Case presentation

A 29-year-old Burmese woman with human immunodeficiency virus (HIV) infection who had been diagnosed with pulmonary tuberculosis was initially treated with isoniazid 300 mg/day, rifampicin 450 mg/day, pyrazinamide (unknown dosage), and ethambutol 800 mg/day for 2 months. After a 2-month course of this four-drug regimen, pyrazinamide was discontinued. Five months after the initiation of ATT, the patient developed pain, redness, and blurred vision in her right eye. Two weeks later, her right eye became no light perception and exhibited proptosis with the presence of purulent discharge. Orbital computed tomography (CT) showed large intraconal, periorbital, and preseptal abscesses. She was then admitted to the primary care hospital and was treated for orbital cellulitis with a 1-g ceftriaxone infusion twice daily. Oral ciprofloxacin 500 mg twice daily was added 4 days later. Tobramycin eye drops 0.3% four times daily and tobramycin eye ointment twice daily were given.

**Fig. 1:**
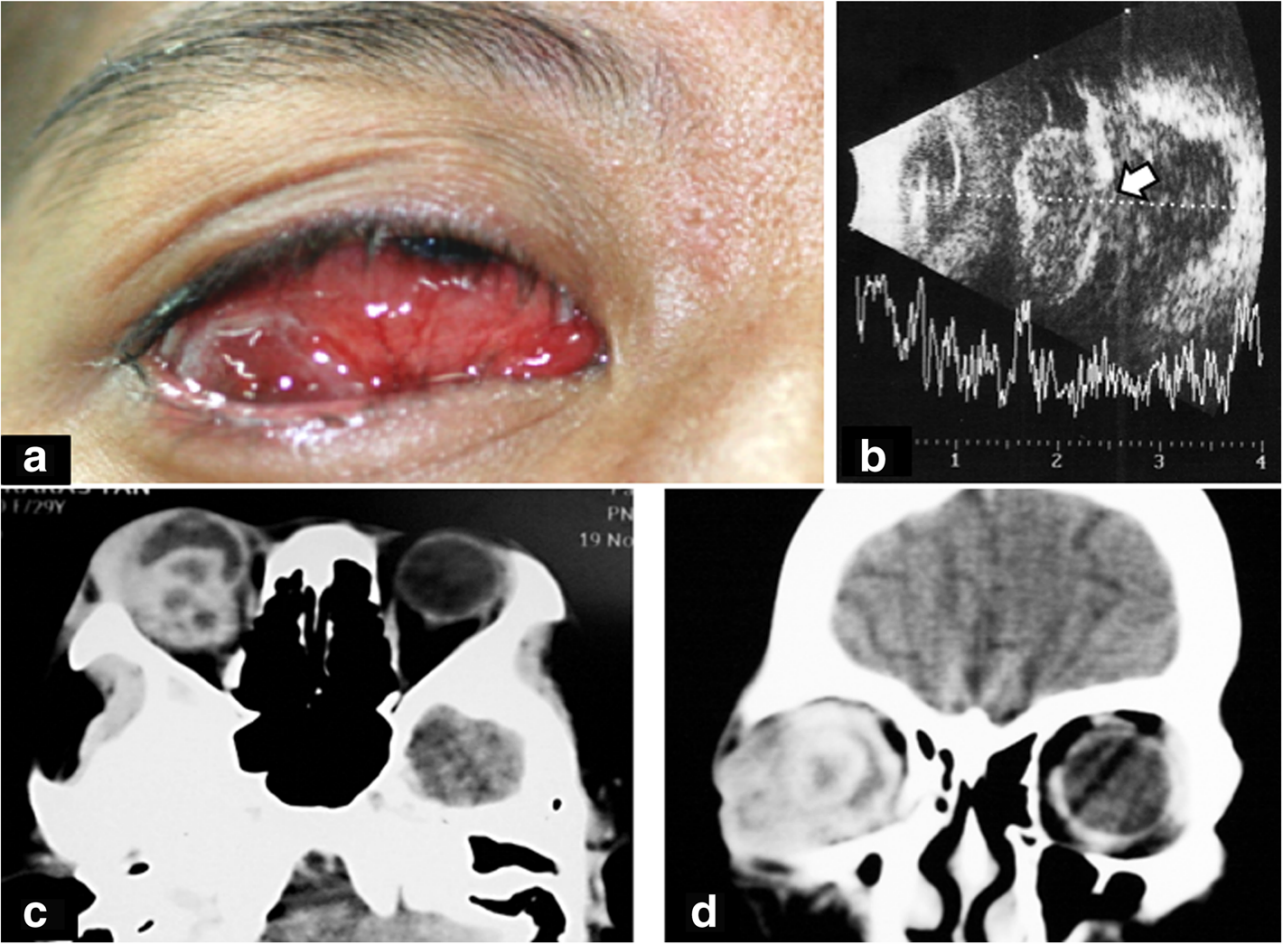
**a** A large subconjunctival abscess is shown in the right eye inferotemporally with purulent discharge and chemosis. **b** B-scan ultrasonogram of the right eye shows a large intraocular mass extending beyond the globe through a scleral defect (*arrow*) into the orbital cavity. **c** and **d** Orbital computed tomographic scan shows in more detail the conjoint intraocular and intraorbital multiloculated, multiple-ring, contrast-enhanced mass

The result of a conjunctival pus swab was positive for 1+ acid-fast bacilli (AFB), and a culture revealed clusters of *Staphylococcus* spp. Despite an escalation of ATT and antibiotics for 1 week, the patient’s clinical condition continued to worsen. She was then referred to our institution, Siriraj Hospital, Thailand’s largest university-based tertiary referral center. Upon arrival, her right eye showed marked chemosis with a large subconjunctival inferotemporal abscess (Fig. 1a). She had 3+ anterior chamber cells, mutton-fat keratic precipitates, ectropion uvea, and iris neovascularization. Exophthalmometry at 104-mm base showed a protrusion of the right eye of 20 mm, compared with 13 mm in the left eye. The right lateral gaze and down gaze of her right eye were limited at 5 and 10 degrees, respectively. A dilated fundus examination showed a dense vitreous hemorrhage. An unclear subretinal lesion was identified. Her left eye was unremarkable. B-scan ultrasonography of her right eye showed an irregular globe contour and inhomogeneous vitreous echogenicity. A 13.3 mm×15 mm mass with a high initial A-scan spike and low to moderate internal reflectivity was identified underneath a high A-scan spike membrane-like lesion that extended beyond the globe into the orbital cavity (Fig. 1b).

**Fig. 2:**
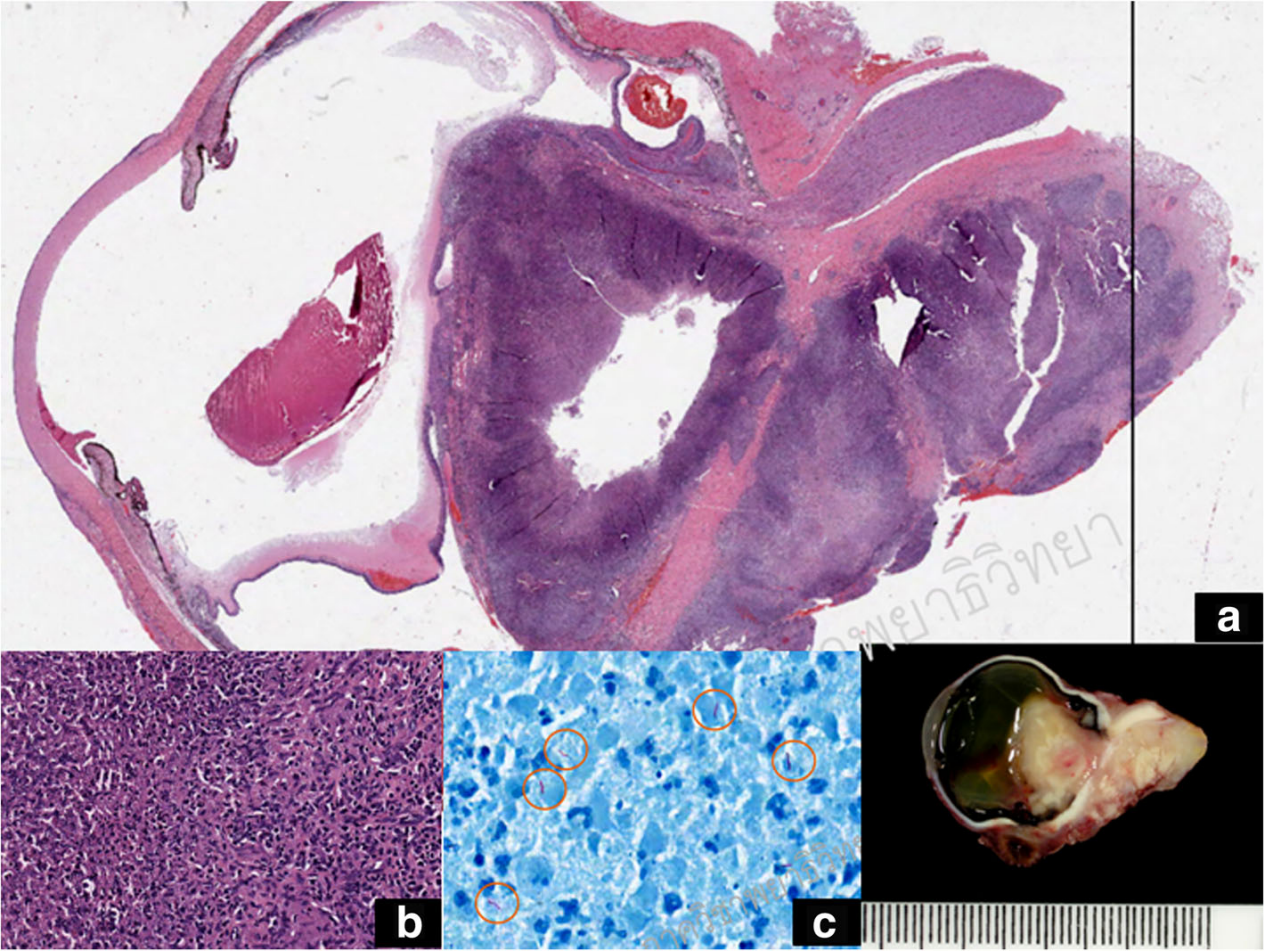
**a** A histopathological section of the right eyeball shows an ill-defined, soft, grayish white mass with central necrosis extending from the vitreous cavity into a retrobulbar region, attached to sclera and closed to the optic nerve. **b** Microscopic findings revealed granulomatous inflammation with accumulations of predominantly mononuclear cells. **c** Acid-fast bacilli are shown within *orange circles*

Orbital CT was repeated, which showed an intraocular multiloculated ring contrast-enhanced mass that extended to the right orbit (Fig. 1c and d). The optic nerve, lateral rectus muscle, and inferior rectus muscle of the right eye were enlarged. The conjunctival pus swab was repeated, which stained positive for AFB. The patient’s CD4 cell count was 123 cells/mm^3^. An infectious disease (ID) specialist immediately ordered second-line ATT (750-mg amikacin infusion, 500-mg levofloxacin infusion, oral clarithromycin 1 g/day, and *para*-aminosalicylic acid 8 g/day). With the fact that the infection was intense and was not responding to conventional ATT, despite the previous 5 months of ATT treatment. The risk of progression to the central nervous system was weighed against preservation of the eyeball. With a discussion between the patient and the medical team, a decision was made to perform subtotal orbital exenteration. A large number of scleral and intramuscular tuberculomas were identified. One large subretinal abscess (14 mm×10 mm×10 mm) was present that extended to the orbital cavity through a scleral perforation. Pathological study of exenterated tissue revealed chronic granulomatous inflammation and caseous necrosis, which was positive for AFB stain and compatible with mycobacterial infection (Fig. 2). Cultured tissue was negative for mycobacterium. Three months later, the result of a conjunctival pus swab culture was positive for drug-susceptible Mtb. The ID specialist recommended continuation of the second-line oral ATT for 18 more months. There was no sign of recurrent tuberculous mycobacterium infection around the exenterated wound at the patient’s last visit, the 6-month follow-up, at Siriraj Hospital. After that visit, she was transferred back to her primary care hospital to complete her course of treatment. Highly active antiretroviral therapy, or HAART, was initiated afterward at the primary care hospital according to the health care policy in Thailand.

## Discussion

Reports of tuberculous panophthalmitis and/or orbital tuberculosis with or without orbital abscess are infrequent. As described in Table 1, many patients have either panophthalmitis or orbital infection, unlike our patient, who had extensive panophthalmitis and orbital abscesses despite 5 months of conventional ATT. Our patient’s presentation led to a high suspicion of drug-resistant tuberculosis. In a study done in China, researchers reported that the prevalence of any resistance to first-line drugs was 33.2% and that the prevalence of multidrug-resistant tuberculosis (MDR-TB) was 5.7% [3]. Wilson and Tsukayama reported risk factors for the development and perpetuation of drug-resistant Mtb, as shown in Table 2 [4]. Our patient had drug-susceptible Mtb; thus, drug resistance was not responsible for the poor treatment outcome in her case. Poor ATT compliance was likely the main causative factor. Directly observed treatment, short course (DOTS), especially with an individualized treatment regimen, is considered a successful method to improve cure rate and treatment completion in many centers [5]; moreover, DOTS can also prevent the development of drug resistance [6]. Statistical analysis to evaluate associated risk factors for developing tuberculous mycobacterial orbital abscess and/or panophthalmitis and visual prognosis has not been achievable, owing to the small number of cases in this patient population. For patients with HIV infection, debate continues regarding the ideal timing of HAART initiation and concomitant administration with ATT. The advantages of early HAART administration include higher cure rates, reduced risk of relapse, reduced risk of other HIV-associated opportunistic infections, and lower mortality rates. On the other hand, the disadvantages of early HAART initiation include potential drug interaction with rifampicin, thus limiting coadministration of selected protease inhibitors. This could result in cumulative toxicity, therapeutic failure, and risk of immune reconstitution inflammatory syndrome [2, 7]. Our patient had not received HAART until almost 1 year after she was first diagnosed with EPTB infection.

**Table 1:** Reported cases of *Mycobacterium tuberculosis* panophthalmitis, orbital abscess, orbital tuberculoma, and orbital apex syndrome with wide varieties in age range, treatment outcomes, and visual prognosis

Patient	Age (years)	Sex	Nationality	Health status	Ocular diagnosis	Initial VA	Final VA	Surgical treatment	References
1	29	F	Burmese	HIV	Panophthalmitis with orbital abscess	NPL	–	Subtotal exenteration	Our patient
2	73	M	N/A	Healthy	Panophthalmitis	6/60	–	Enucleation	[8]
3	14	M	N/A	N/A	Panophthalmitis	N/A	N/A	N/A	[9]
4	59	F	Indian	Healthy	Orbital tuberculoma	No visual loss	No visual loss	–	[10]
5	78	F	African	Healthy	Orbital tuberculoma	No visual loss	No visual loss	Anterior orbitotomy	[10]
6	12	F	N/A	N/A	Panophthalmitis	NPL	–	Enucleation	[11]
7	15	F	Indian	N/A	Orbital abscess	6/6	6/6	Abscess drainage	[12]
8	86	F	Caucasian	N/A	Orbital tuberculoma	Blind	Blind	–	[13]
9	6	M	Indian	N/A	Orbital abscess	6/6	6/6	FNA	[14]
10	16	F	Afro-Caribbean	N/A	Orbital apex syndrome	6/190	PL	–	[15]
11	7	F	N/A	Healthy	Orbital abscess	6/6	N/A	Pus evacuation	[16]
12	29	N/A	N/A	Healthy	Orbital mass	N/A	N/A	–	[17]
13	1	N/A	Nigerian	N/A	Panophthalmitis	N/A	–	Enucleation	[18]
14	27	M	Indian	HIV	Orbital abscess	NPL	NPL	–	[19]

*Abbreviations: VA* Visual acuity, *F* Female, *M* Male, *NPL* No perception of light, *PL* Light perception, *HIV* human immunodeficiency virus infected, *FNA* Fine-needle aspiration, *N/A* Not available

**Table 2:** Risk factors for the development of drug resistance to *Mycobacterium tuberculosis* as reported by Wilson and Tsukayama [4]

Previous treatment for Mtb
Prolonged hospitalization (in Mtb-endemic regions)
human immunodeficiency virus coinfection
Inappropriate prescribing of combination ATT (incorrect drug selection, dosing, and improper dispensing)
Lack of directly observed therapy use during therapy and subsequent patient noncompliance with prescribed therapy
Lack of sustainable drug availability to patients (for example, second-line drugs for drug-resistant tuberculosis) through an inadequate pharmaceutical supply chain or failure to provide free treatment
Overuse of fluoroquinolones in other non-Mtb respiratory infection syndromes that propagates fluoroquinolone-resistant Mtb
Delays in diagnosing drug-resistant Mtb

*Abbreviations*: *Mtb* Mycobacterium tuberculosis, *ATT* Antituberculous therapy

## Conclusions

Close and careful observation of patients undergoing ATT is absolutely essential. Poor compliance is one of the main risk factors for developing MDR-TB. Even in cases where there is no drug resistance, poor compliance can alter treatment outcome and prognosis, as evidenced by the course and outcome of our patient.
